# Spatio-temporal electroencephalographic power distribution in experimental pigs receiving propofol

**DOI:** 10.1371/journal.pone.0303146

**Published:** 2024-05-14

**Authors:** Alessandro Mirra, Darren Hight, Claudia Spadavecchia, Olivier Louis Levionnois

**Affiliations:** 1 Vetsuisse Faculty, Department of Clinical Veterinary Medicine, Anesthesiology and Pain Therapy Section, University of Bern, Bern, Switzerland; 2 Department of Anesthesiology and Pain Medicine, Inselspital, Bern University Hospital, University of Bern, Bern, Switzerland; Georgia State University, UNITED STATES

## Abstract

**Introduction:**

When assessing the spatio-temporal distribution of electroencephalographic (EEG) activity, characteristic patterns have been identified for several anesthetic drugs in humans. A shift in EEG power from the occipital to the prefrontal regions has been widely observed during anesthesia induction. This has been called “anteriorization” and has been correlated with loss of consciousness in humans. The spatio-temporal distribution of EEG spectral power in pigs and its modulation by anesthetics have not been described previously. The aim of the present study was to analyze EEG power across an anterior-posterior axis in pigs receiving increasing doses of propofol to 1) characterize the region of highest EEG power during wakefulness, 2) depict its spatio-temporal modification during propofol infusion, and 3) determine the region demonstrating the most significant modulations across different doses administered.

**Materials and methods:**

Six pigs with a body weight of 33.3 ± 3.6 kg and aged 11.3 ± 0.5 weeks were included in a prospective experimental study. Electroencephalographic activity was collected at the occipital, parietal and prefrontal regions at increasing doses of propofol (starting at 10 mg kg^-1^ h^-1^ and increasing it by 10 mg kg^-1^ h^-1^ every 15 minutes). The EEG power was assessed using a generalized linear mixed model in which propofol doses and regions were treated as fixed effects, whereas pig was used as a random effect. Pairwise comparisons of marginal linear predictions were used to assess the change in power when the specific propofol dose (or region) was considered.

**Results:**

During both wakefulness and propofol infusion, the highest EEG power was located in the prefrontal region (p<0.001). The EEG power, both total and for each frequency band, mostly followed the same pattern, increasing from awake until propofol 20 mg kg^-1^ h^-1^ and then decreasing at propofol 30 mg kg^-1^ h^-1^. The region showing the strongest differences in EEG power across propofol doses was the prefrontal.

**Conclusion:**

In juvenile pigs receiving increasing doses of propofol, the prefrontal region showed the highest EEG power both during wakefulness and propofol administration and was the area in which the largest frequency-band specific variations were observed across different anesthetic doses. The assessment of the spectral EEG activity at this region could be favorable to distinguish DoA levels in pigs.

## Introduction

The evaluation of the raw electroencephalographic (EEG) activity and its spatio-temporal distribution is of paramount importance for understanding the effect and the mechanism of action of general anesthetics [1,2]. Through its analysis, characteristic patterns (signatures), related to molecular and neural circuits have been identified in humans for several anesthetic drugs [2,3], allowing a more accurate assessment of dose- and time-dependent effects on brain activity compared to algorithm-based indexes [4,5].

Predominant EEG power has been shown to be located in the occipital brain region in awake humans [6]. During the administration of gamma-aminobutyric acid (GABA) receptor agonists (e.g., propofol, isoflurane), a power shift toward the frontal region has been observed, particularly in the alpha frequency band. This phenomenon is named anteriorization [7,8] and has been associated with unconsciousness in humans [5,6]. Together with the convenient lack of hair, this has probably contributed to the development of depth of anesthesia (DoA) monitors analyzing the EEG signal from frontal regions alone.

If the EEG power spatio-temporal distribution following the administration of various anesthetics has been reported in humans, almost no information is available in veterinary medicine, particularly in pigs [9–11]. Similar EEG monitoring approaches to those used in humans have been applied in pigs undergoing experimental procedures [12], including EEG-based DoA monitors developed for and based on human data. However, many studies have demonstrated their inaccuracy in correctly assessing unconsciousness in these animals, probably due to the underlying anatomical and neurophysiological differences [13,14].

The characterization of the EEG power spatio-temporal distribution in pigs during wakefulness and while receiving anesthetic drugs is essential for the development of species-specific methodologies for detecting unconsciousness and differentiating anesthetic levels. This would allow clinicians to ensure animal welfare during experimental procedures, particularly when invasive surgeries are performed and when neuromuscular blocking agents are administered [15]. Moreover, it would constitute an important and novel opportunity to explore mechanisms of action of general anesthetics on the central nervous system.

The present study aimed to analyze the EEG power across the anterior-posterior axis in pigs receiving increasing doses of propofol to 1) identify the region of highest EEG power during wakefulness, 2) depict the EEG power spatio-temporal modification during propofol infusion, and 3) determine the anatomical region demonstrating the most significant modulations across administered doses of propofol.

We hypothesized that, as in humans, the occipital region would show the greatest EEG power during wakefulness and that a shift toward the frontal region would occur during the administration of increasing propofol doses. Moreover, we hypothesized that the frontal region, supposed to have the highest power, would also be the most appropriate to distinguish EEG spectral differences among anesthetic levels.

## Methods

The present trial was part of a larger study aiming at investigating recovery characteristics in pigs. Ethical permission was obtained from the Committee for Animal Experiments of the Canton of Bern, Switzerland (Protocol Number: 32015). Sample size was calculated for the recovery study and not specifically for this trial. However, an a-posteriori sample size calculation using data collected from our group during the experiment (and not included in the present study) [16] confirmed that 6 animals would be needed to detect a significant difference between two different anesthetic levels, based on the following: EEG power in the delta frequency band at surgical level of anesthesia 17.9 (1.7) dB [mean ± standard deviation (SD)]; EEG power in the delta frequency band at early recovery 13.9 (2.1); calculated effect size 2.07; two tails; power 0.95; alpha 0.05 (Wilcoxon signed-rank test; G*Power 3.1.9.6 2020).

### Animals

This prospective experimental study involved six pigs (phenotype Edelschwein) of both sexes (four females, two males) with a body weight of 33.3 ± 3.6 kg and aged 11.3 ± 0.5 weeks.

Pigs were collected in groups of at least two animals from the farm of origin, between two and ten days before the experimental trial. The animals were housed in single boxes in the animal facility of the University of Bern, Vetsuisse Faculty. Visual and auditory contact between animals was always allowed.

### Instrumentation

**Fig 1:**
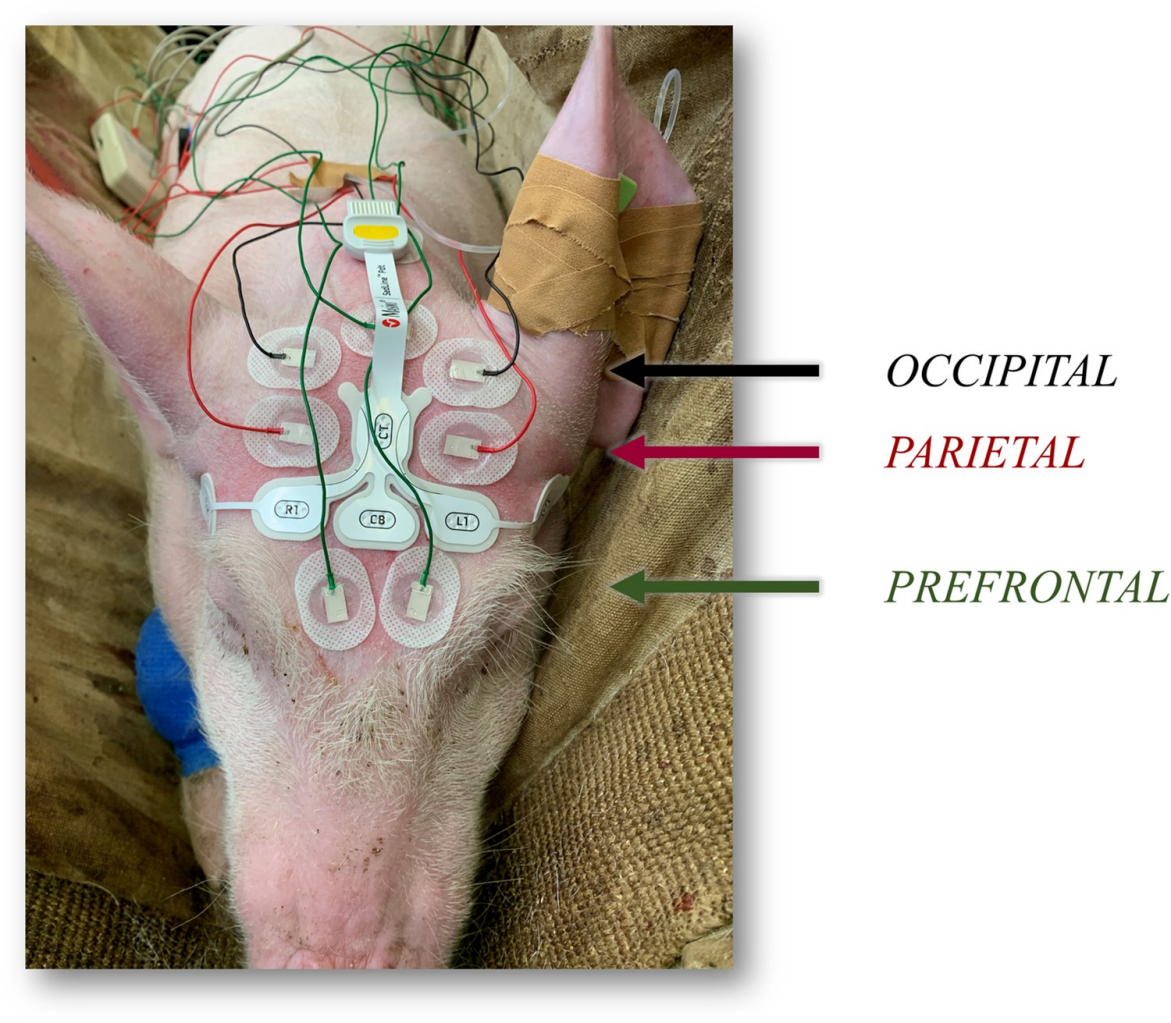
Positioning of the RD SedLine pediatric EEG-sensor and six further surface electrodes for the recording of the electroencephalographic (EEG) activity. The main RD SedLine electrodes (L2, L1, R1, R2, from left to right) were placed on a transverse line over the frontal bone, keeping their rostral border on an imaginary line running between the lateral canthi of the eyes. Six additional surface EEG electrodes were used. Four of them were positioned caudally to the RD SedLine EEG-sensor, on the same sagittal line of L1 and R1: Two just rostral to the caudal margin of the occipital bone (“occipital”) and two in between these and the RD SedLine EEG-sensor (“parietal”). The last two surface electrodes were positioned between the eyes (“prefrontal”).

On the experimental days, pigs were brought to the experimental room and left undisturbed for 30–60 minutes. Then, they were placed into a sling for instrumentation. A local anesthetic cream (EMLA 5%, Anesderm, Pierre Fabre, Switzerland) was applied over the two ears and on the tail for at least 45 minutes before placing venous (auricular) and arterial (auricular or coccygeal) catheters. The skin over the skull was then prepared, as previously described [16]. Briefly, the area between the frontal and the occipital bone was clipped, cleaned and shaved. Thereafter, it was rubbed with an abrasive paper (Red Dot Trace Prep, 3 M Health Care, Canada) and defatted (Benzinum Medicinale, Hänseler AG, Switzerland). Once the skin was dry, the RD SedLine pediatric EEG-sensor was positioned as previously reported [16]. The electrodes were placed on a transverse line over the frontal bone, keeping their rostral border on an imaginary line running between the lateral canthi of the eyes (L2, L1, R1, R2, from left to right; Fig 1). The central CB (ground) and the caudal CT (reference) electrodes were placed on the mid-sagittal line. Six additional surface EEG electrodes (Ambu® Neuroline 715; Ambu, Ballerup, Denmark) were used (Fig 1). Four of them were positioned on the same sagittal line than L1 and R1: two just rostral to the caudal margin of the occipital bone (“occipital”) and two in the middle between these and the RD SedLine EEG-sensor (“parietal”). The last two surface electrodes were positioned between the eyes (“prefrontal”). The signal collected from these electrodes was sent to an amplifier (EEG100c, Biopac Systems Inc, California, USA) and a data acquisition module (MP160, Biopac Systems Inc, California, USA). The SedLine monitor continuously calculated and displayed the EEG suppression ratio (SR) using a proprietary algorithm that measures the percentage of time within a moving window during which brain electrical activity is suppressed. Only signals recorded by the Biopac System were analyzed for the purpose of the present study.

### Treatment

Propofol (Propofol 1% MCT, Fresenius Kabi AG, Switzerland) was started as an intravenous (IV) infusion at 10 mg kg^-1^ h^-1^, and increased by 10 mg kg^-1^ h^-1^ every 15 minutes. Oxygen supplementation was always provided (face mask). Endotracheal intubation was performed when deemed appropriate by an experienced anesthetist (AM); afterwards, volume-controlled mechanical ventilation was started (15 ml kg^-1^; respiratory rate adjusted targeting an end-tidal carbon dioxide (EtCO_2_) between 35 and 45 mmHg). When the SR reached values above 80% for 10 consecutive minutes, the propofol infusion was stopped and the pig allowed to recover. Data collection was performed for the following 90 minutes or until full recovery was reached. Then, euthanasia was performed with pentobarbital IV (150 mg kg^-1^).

### Data collection

Electroencephalographic data were recorded from both sides of the skull, while analysis was performed only on data collected from the right side. Bipolar derivations were used. The reference electrode was placed on the sagittal midline caudally to the occipital electrodes (Fig 1). In our analysis, for the bipolar occipital signal the reference and occipital electrodes were used, for the bipolar parietal signal the occipital and parietal electrodes, and for the bipolar prefrontal signal the parietal and prefrontal electrodes (Fig 1).

The EEG signals were recorded using the Biopac related software (AcqKnowledge®; Biopac Systems Inc, California, USA). Sampling rate was 500 Hz. Data were then analyzed in Matlab (The MathWorks Inc., Natick, Massachusetts, USA). A high-pass filter set at 0.1 Hz (Butterworth, 3rd order, zero-phase distortion) was applied to allow supplementary suppression detection due to removal of low frequency drifting. Five minutes epochs were extracted and analyzed during stages when the pigs were awake, and at the end of each propofol infusion level (the 5 minutes preceding the next increase). Epochs including periods of EEG suppression were excluded to avoid artifacts when creating power spectra. For this purpose, the EEG trace of each pig was visually inspected by one investigator (DH); periods of EEG suppression were defined as those having an amplitude lower than approximately 5 μV and longer than approximately one second. To simplify analysis and avoid analyzing EEGs from a smaller number of animals, the lowest propofol dosage at which EEG suppression occurred in at least one animal was determined and all the data collected at this level and above were excluded. From this analysis, it resulted that none of the individual EEGs displayed suppression up to a propofol infusion rate of 30 mg kg^-1^ h^-1^. Thus, the following time points were evaluated: A) Awake: no propofol; B) Propofol 10: propofol 10 mg kg^-1^ h^-1^; C) Propofol 20: propofol 20 mg kg^-1^ h^-1^; D) Propofol 30: propofol 30 mg kg^-1^ h^-1^. Subsequently, power spectra were created using the discrete fast Fourier transform using the “spectrogram.m” algorithm on non-overlapping windows of 4 seconds length, with the number of Discrete Fourier Transform points set to 1000, yielding a frequency resolution of 0.5 Hz.

### Statistical analysis

For each subject, median EEG power was calculated for five specific frequency bands: 0.1–4 Hz (delta), 5–8 Hz (theta), 9–12 Hz (alpha), 13–25 Hz (beta-1), and 26–35 Hz (beta-2 or gamma). Median power for each frequency band and pig was extracted from the spectra and analyzed. The final data set was longitudinal with repeated measures for frequency bands, propofol doses, and anatomical regions. To account for these factors, data were examined using a generalized linear mixed model in which propofol doses or regions were treated as fixed effects, whereas pigs were used as random effect. Restricted maximum likelihood was used as link function to provide unbiased estimates of the variance componence of the random effects. The statistical significance level (p) was set at 0.01 after Bonferroni correction. To assess significant changes in outcomes at different propofol doses or scalp regions, statistically significant differences between predictive mean margins—predictions of the fitted model at fixed propofol levels or scalp regions according to the specific model—were assessed by pairwise comparisons of such predictive mean margins (contrast of marginal means). Marginal plots were then drawn plotting scalp regions or propofol doses against predictive marginal means. The difference between EEG power values (D) is reported.

Moreover, the difference between prefrontal and occipital power for the different frequency bands was calculated for each time point. Normality was assessed using the Shapiro-Wilk test, and differences over time using the One Way Repeated Measures Analysis of Variance followed by the Holm-Sidak test. The statistical significance level was set at 0.05. Statistical analyses were performed using Stata17 (StataCorp., College Station, TX, USA) and SigmaPlot (Version 15; Systat Software Inc., CA, USA).

## Results

**Fig 2:**
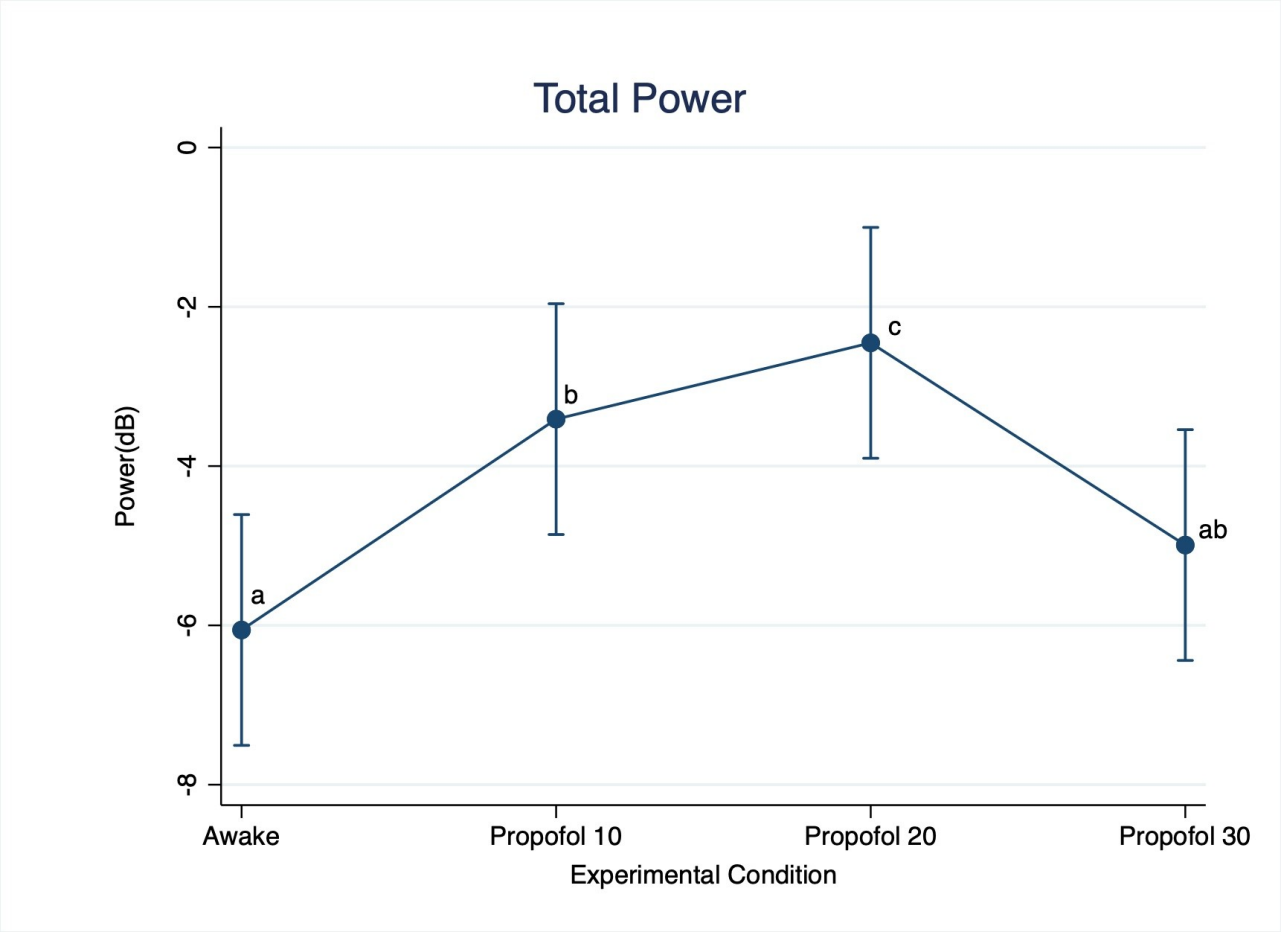
Change in the total electroencephalographic power with increasing doses of propofol. For statistical analysis, a mixed model was used with propofol doses and frequency bands as fixed effects and pig as random effect. Statistical significance (p < 0.01) is indicated with letters: Equal letters between two mean values indicate the absence of statistically significant difference.

Total EEG power was statistically lower at Awake compared to Propofol 10 (p < 0.001; D = 2.65 dB) and Propofol 20 (p < 0.001; D = 3.62 dB), and it was significantly lower at Propofol 30 compared to Propofol 20 (p < 0.001; D = 2.54 dB) (Fig 2).

Furthermore, total power was always significantly higher at the Prefrontal compared to both the Parietal (p<0.001) and Occipital (p<0.001) regions. These results were also confirmed when a subgroup analysis was performed for each frequency band and for each time point (S1 and S2 Appendices).

**Fig 3:**
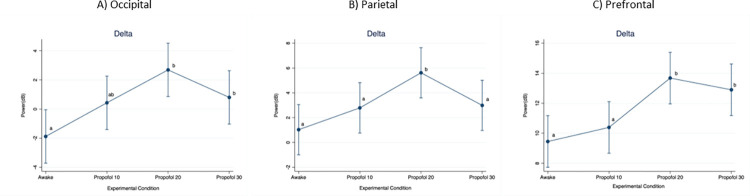
Mean (95%CI) electroencephalographic power over the occipital, parietal and prefrontal regions, at increasing propofol doses. Frequency band: Delta. For statistical analysis, a mixed model was used with propofol dose as fixed effect and pig as random effect. Statistical significance (p < 0.01) is indicated with letters: Equal letters between two mean values indicate the absence of statistically significant difference.

When considering the delta frequency band (Fig 3A–3C), power in the occipital region was significantly lower at Awake compared to Propofol 20 (p < 0.001; D = 4.57 dB) and Propofol 30 (p = 0.005; D = 2.68 dB). In the parietal region, power was significantly lower at Awake compared to Propofol 20 (p < 0.001; D = 4.59 dB) and significantly higher at Propofol 20 compared to Propofol 10 (p < 0.001; D = 2.83 dB) and Propofol 30 (p = 0.001; D = 2.63 dB). In the prefrontal region, power was significantly lower at Awake compared to Propofol 20 (p < 0.001; D = 4.23 dB) and Propofol 30 (p < 0.001; D = 3.45 dB), and at Propofol 10 compared to Propofol 20 (p < 0.001; D = 3.29) and Propofol 30 (p = 0.001; D = 2.51 dB).

**Fig 4:**
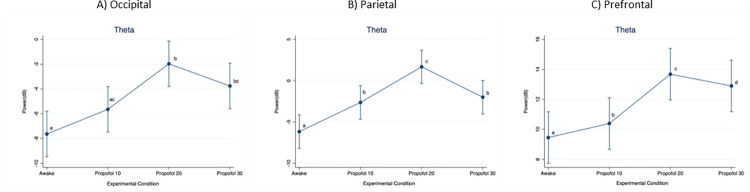
Mean (95%CI) electroencephalographic power over the occipital, parietal and prefrontal regions, at increasing propofol doses. Frequency band: Theta. For statistical analysis, a mixed model was used with propofol dose as fixed effect and pig as random effect. Statistical significance (p < 0.01) is indicated with letters: Equal letters between two mean values indicate the absence of statistically significant difference.

When considering the theta frequency band (Fig 4A–4C), power in the occipital region was significantly lower at Awake compared to Propofol 20 (p < 0.001; D = 5.66 dB) and Propofol 30 (p < 0.001; D = 3.87 dB), as well as at Propofol 10 compared to Propofol 20 (p < 0.001; D = 3.67 dB).

In the parietal region, power was significantly lower at Awake compared to Propofol 10 (p < 0.001; D = 3.53 dB), Propofol 20 (p < 0.001; D = 7.86 dB) and Propofol 30 (p < 0.001; D = 4.17 dB). Moreover, it was significantly higher at Propofol 20 compared to Propofol 10 (p < 0.001; D = 4.31) and Propofol 30 (p < 0.001; D = 3.68 dB). In the prefrontal region, power was significantly lower at Awake compared to Propofol 10 (p < 0.001; D = 4.17 dB), Propofol 20 (p < 0.001; D = 9.18 dB) and Propofol 30 (p < 0.001; D = 6.29). Moreover, it was significantly higher at Propofol 20 compared to Propofol 10 (p < 0.001; D = 5.01) and Propofol 30 (p < 0.001; D = 2.89 dB), and at Propofol 30 compared to Propofol 10 (p = 0.006; D = 2.11).

**Fig 5:**
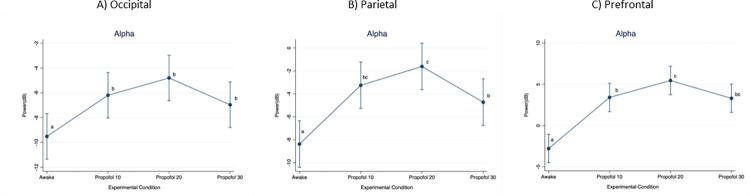
Mean (95%CI) electroencephalographic power over the occipital, parietal and prefrontal regions, at increasing propofol doses. Frequency band: Alpha. For statistical analysis, a mixed model was used with propofol dose as fixed effect and pig as random effect. Statistical significance (p < 0.01) is indicated with letters: Equal letters between two mean values indicate the absence of statistically significant difference.

When considering the alpha frequency band (Fig 5A–5C), power in the occipital region was significantly lower at Awake compared to Propofol 10 (p = 0.001; D = 3.31 dB), Propofol 20 (p < 0.001; D = 4.71 dB) and Propofol 30 (p = 0.008; D = 2.55 dB). In the parietal region, power was significantly lower at Awake compared to Propofol 10 (p < 0.001; D = 5.12 dB), Propofol 20 (p < 0.001; D = 6.76 dB) and Propofol 30 (p < 0.001; D = 3.65 dB), as well as at Propofol 30 compared to Propofol 20 (p < 0.001; D = 3.11). In the prefrontal region, power was significantly lower at Awake compared to Propofol 10 (p < 0.001; D = 6.21 dB), Propofol 20 (p < 0.001; D = 8.25 dB) and Propofol 30 (p < 0.001; D = 6.10 dB), as well as at Propofol 10 compared to Propofol 20 (p = 0.008; 2.04 dB).

**Fig 6:**
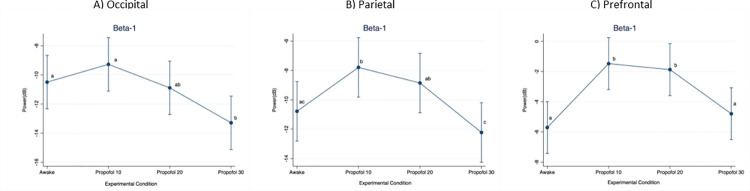
Mean (95%CI) electroencephalographic power over the occipital, parietal and prefrontal regions, at increasing propofol doses. Frequency band: Beta-1. For statistical analysis, a mixed model was used with propofol dose as fixed effect and pig as random effect. Statistical significance (p < 0.01) is indicated with letters: Equal letters between two mean values indicate the absence of statistically significant difference.

When considering the beta-1 frequency band (Fig 6A–6C), power in the occipital region was significantly lower at Propofol 30 compared to Awake (p = 0.004; D = 2.79 dB) and Propofol 10 (p < 0.001; D = 4.02 dB). In the parietal region, power was significantly higher at Propofol 10 compared to Awake (p < 0.001; D = 2.99 dB) and Propofol 30 (p < 0.001; D = 4.43 dB). Moreover, it was significantly lower at Propofol 30 compared to Propofol 20 (p < 0.001; D = 3.37 dB). In the prefrontal region, power was significantly lower at Awake compared to Propofol 10 (p < 0.001; D = 4.23 dB) and Propofol 20 (p < 0.001; D = 3.83 dB), as well as at Propofol 30 compared to Propofol 10 (p < 0.001; D = 3.32 dB) and Propofol 20 (p < 0.001; D = 2.92 dB).

**Fig 7:**
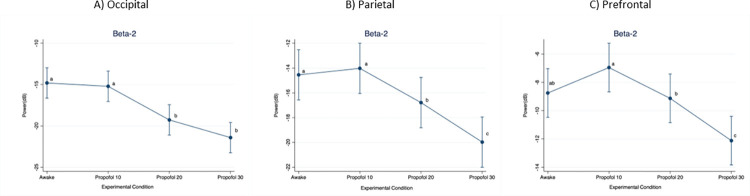
Mean (95%CI) electroencephalographic power over the occipital, parietal and prefrontal regions, at increasing propofol doses. Frequency band: Beta-2. For statistical analysis, a mixed model was used with propofol dose as fixed effect and pig as random effect. Statistical significance (p < 0.01) is indicated with letters: Equal letters between two mean values indicate the absence of statistically significant difference.

When considering the beta-2 frequency band (Fig 7A–7C), power in the occipital region was significantly higher at Awake compared to Propofol 20 (p < 0.001; D = 4.47 dB) and Propofol 30 (p < 0.001; D = 6.61 dB). Moreover, it was significantly higher at Propofol 10 compared to Propofol 20 (p < 0.001; D = 4.06 dB) and Propofol 30 (p < 0.001; D = 6.20 dB).

In the parietal region, power was significantly higher at Awake compared to Propofol 20 (p = 0.006; D = 2.24 dB) and Propofol 30 (p < 0.001; D = 5.42 dB). Moreover, it was significantly higher at Propofol 10 compared to Propofol 20 (p = 0.001; D = 2.76 dB) and Propofol 30 (p < 0.001; D = 5.95 dB), as well as at Propofol 20 compared to Propofol 30 (p < 0.001; D = 3.19 dB).

In the prefrontal region, power was significantly lower at Propofol 30 compared to Awake (p < 0.001; D = 3.37 dB), Propofol 10 (p < 0.001; D = 5.16 dB) and Propofol 20 (p < 0.001; D = 2.99 dB). Moreover, it was significantly higher at Propofol 10 compared to Propofol 20 (p = 0.004; D = 2.18 dB).

Examples of raw EEG traces and the spectrograms have been reported in the supplemental materials (S3 and S4 Appendices).

A significantly higher difference between prefrontal and occipital power was found in the theta frequency band at Propofol 20 (p = 0.001) and Propofol 30 (p = 0.026) compared to Awake. A significant higher difference was also found in the alpha and beta frequency bands at Propofol 10 (p = 0.005 and 0.017), Propofol 20 (p = 0.001 and 0.004) and Propofol 30 (p = 0.002 and 0.008), as well as in the gamma frequency band at Propofol 20 (p = 0.042) compared to Awake. No other significant differences were found. Further information can be found in the supplemental materials (S5 Appendix).

## Discussion

The results of the present study showed that, contrary to our hypothesis, the highest EEG power was located in the prefrontal and not in the occipital region during wakefulness. This was also observed during propofol administration. The EEG power followed a similar pattern in almost all frequency bands, increasing from awake until Propofol 20, and then decreasing at Propofol 30. The region showing the most significant differences in EEG power across time points was the prefrontal.

In humans, loss of consciousness is generally identified via loss of patient response to commands, and it is often used as target in studies investigating EEG power modification during propofol administration [3,8]. Given that this technique is not applicable in animals, other endpoints need to be used to identify a deep hypnotic state. In the present study, clinical parameters were excluded due to the great variability previously observed [17]. The occurrence of EEG suppression was rather targeted, and data collected after its supposed onset were excluded. Indeed, spectral analyses does not yield meaningful results if performed when EEG suppression is present, as the EEG signal normally becomes strongly non-stationary (high-amplitude bursts alternating with periods of low-amplitude silence) [18].

In awake humans with eyes closed, predominant EEG power is present in the occipital region. During propofol sedation, a frontal shift is observed (anteriorization). In particular, an increase in the alpha and beta power in the frontal region accompanied by a decrease in alpha power in the occipital region occurs [8,19,20]. With higher anesthetic dose, an increase in delta power (EEG slow waves) in both occipital and frontal regions follows [8,19,20].

Our results do not support the presence of anteriorization in pigs receiving propofol, being the EEG power always higher in the prefrontal region, independently on frequency band considered and dose administered. Despite this, a larger alpha and beta power increase in the prefrontal region compared to the occipital region was noticed when propofol infusion was started (Propofol 10). At higher infusion rates (Propofol 20 and Propofol 30), a similar power modification was found instead. Future studies using a higher number of electrodes could help to further characterize the EEG spatio-temporal distribution in pigs receiving propofol.

In humans, the occipital alpha peak likely originates from the visual cortex, and has been shown to be higher in subjects with closed compared to open eyes, facilitating identification of power anteriorization [21]. Because the animals cannot be forced to close their eyes while awake, it remains unclear if this may have influenced the absence of occipital alpha dominance observed in the present results. However, despite being weaker, the alpha peak has also been reported in humans with open eyes, especially at a young age [22]. Additionally, some of the subjects of the present study closed their eyes for moderate periods of time during episodes of relaxation before starting the propofol infusion. This has not been specifically recorded and analyzed here, but it may be expected that some degree of increased occipital alpha activity should have been observed if this feature was present in pigs. Finally, detailed comparative anatomy of human and swine brain is lacking, and the electrodes placement may not have captured the activity of the primary visual cortex. Yet, published anatomical studies support the positioning adopted in the present study [23,24].

The age should also be considered for further interpretations. Only juvenile pigs were included here as this is the common age for laboratory pigs to be included in experimental settings. In humans, anteriorization has been shown to start occurring in children of about one year of age [4,25]. The age correspondence between pigs and humans is not known, but it could be that adult animals may display different EEG patterns than observed in the present study. Care should be taken when translating results across pigs of different ages.

The EEG power increased in all frequency bands when propofol was started (Propofol 10) compared to the awake state. Interestingly, at Propofol 20, low frequency bands (delta and theta) continued to significantly rise, alpha stabilized, and both beta-1 and -2 started to decrease. We speculate that this may be associated with an increasing steepness of the aperiodic component of the EEG with a ‘rotation frequency’ located within the alpha to low beta frequency ranges [26]. At Propofol 30, the EEG power decreased in all frequency bands. This resembles what has been observed in humans during anesthesia induction with different drugs (defined as “biphasic effect”), and suggested to be linked with the initiation of loss of consciousness [27]. It is not known if this pattern reflects the same transition in animals, but further studies clarifying this point may be of high relevance. Even if direct feedback cannot be obtained from the animals, the analysis of various clinical and neurophysiological outcomes at increasing doses of anesthetics could allow further insights into their consciousness state.

With regard to other species, a rise in delta and theta power associated with a decrease in beta power has been previously shown in horses receiving increasing doses of halothane [28]. In rats administered a bolus of chloral hydrate, a similar trend in the delta power has been observed, while rather an initial decrease followed by a later increase has been found in higher frequency bands [29]. It may be difficult to compare these results as different methodologies (e.g., monopolar/bipolar EEG recordings) and electrodes placement (e.g., frontal, parietal, occipital) were used.

The results of the present study suggest that the assessment of the EEG power distribution over frequency bands could help differentiating clinically relevant DoA levels in pigs. The knowledge of the drug-specific EEG power distribution (signature) allows a neurophysiology-based assessment of the brain state, giving the opportunity to the anesthetists to recognize baseline patterns, and to identify possible modifications related to changes in DoA levels [30]. The challenge of real-time evaluation of the EEG trace, coupled with the availability of monitors that offer a user-friendly DoA-index, may have contributed to a decline in its investigation. The current availability of monitors facilitating real-time assessment of the EEG power distribution (spectrogram/density spectral array) should promote this methodology that may reveal more appropriate for evaluating DoA, while the adequate approach of interpretation remains to be established.

So far, no veterinary DoA monitors have been developed, but based on our findings, the analysis of the prefrontal region appears appropriate in pigs, as differences in EEG power with increasing propofol doses were most clearly highlighted at this location. This is in line with a recent study in humans that investigated 32 EEG channels and found the frontal F8 and temporal T7 channels to be the most appropriate to discriminate between asleep and awake states [31].

The current study has some additional limitations. The evaluation of the EEG suppression was performed visually by subjectively assessing the presence of flatter EEG periods within the epochs of interest. Since no species-specific algorithm has been defined for pigs, we deemed this approach the most appropriate, as we intended to avoid inadequate calculation of power spectrum. Only six electrodes (three/side) have been used to assess the EEG power spatio-temporal distribution: further studies should be conducted to better characterize it over the whole brain.

## Conclusion

In juvenile pigs receiving increasing doses of propofol, the prefrontal region showed the highest EEG power both during wakefulness and propofol administration and was the area in which the largest frequency-band specific variations were observed across different anesthetic doses. The assessment of the spectral EEG activity at this region could be favorable to distinguish DoA levels in pigs.

## Supporting information

S1 Appendix Mean (95%CI) electroencephalographic power over the occipital, parietal and prefrontal regions, overall and for each frequency band. For statistical analysis, a mixed model was used with regions as fixed effects and pigs as random effects. Statistical significance (p < 0.01) is indicated with letters: Equal letters between two mean values indicate the absence of statistically significant difference. D = difference between EEG power values.
(DOCX)

S2 Appendix Mean (95%CI) electroencephalographic power over the occipital, parietal and prefrontal regions, for each frequency band and for each propofol dose. For statistical analysis, a mixed model was used with regions as fixed effects and pigs as random effects. Statistical significance (p < 0.01) is indicated with letters: Equal letters between two mean values indicate the absence of statistically significant difference. D = difference between EEG power values.
(DOCX)

S3 Appendix Sample 10 second sections of raw electroencephalographic signal from three pigs, all receiving propofol doses of 30 mg kg^-1^ h^-1^, from occipital (blue trace) and prefrontal (orange trace) regions. (DOCX)

S4 Appendix Electroencephalographic spectrogram from three pigs. The signal has been recorded from the right prefrontal electrode. Green vertical line: Start of propofol infusion; red vertical line: End of propofol infusion.
(PDF)

S5 Appendix Difference between prefrontal and occipital electroencephalographic power (in dB), for each time point, pig (identified with letters) and frequency band. (DOCX)
