# Supplementary material for: Spatio-temporal electroencephalographic power distribution in experimental pigs receiving propofol
Source: PLoS One. 2024 May 14;19(5):e0303146. doi: 10.1371/journal.pone.0303146 (PMC11093367; doi:10.1371/journal.pone.0303146)
Supplement: S1 Appendix — For statistical analysis, a mixed model was used with regions as fixed effects and pigs as random effects. Statistical significance (p < 0.01) is indicated with letters: Equal letters between two mean values indicate the absence of statistically significant difference. D = difference between EEG power values. (DOCX) [file pone.0303146.s001.docx]

| **A**  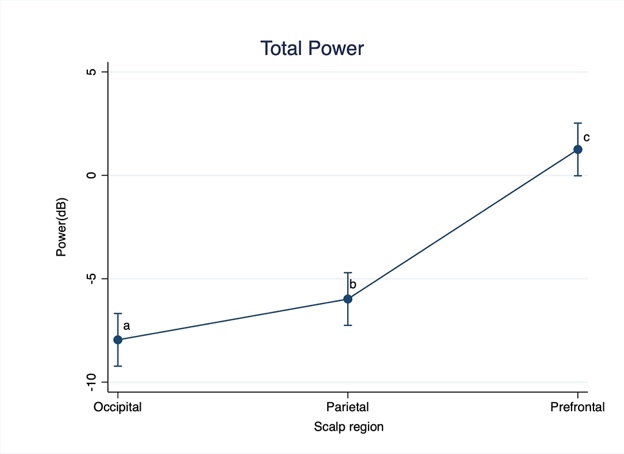  1) Occipital vs. Parietal (D = 1.97 dB, p < 0.001)  2) Occipital vs. Prefrontal (D = 9.20 dB, p < 0.001)  3) Parietal vs. Prefrontal (D = 7.23 dB, p < 0.001) | **B**  **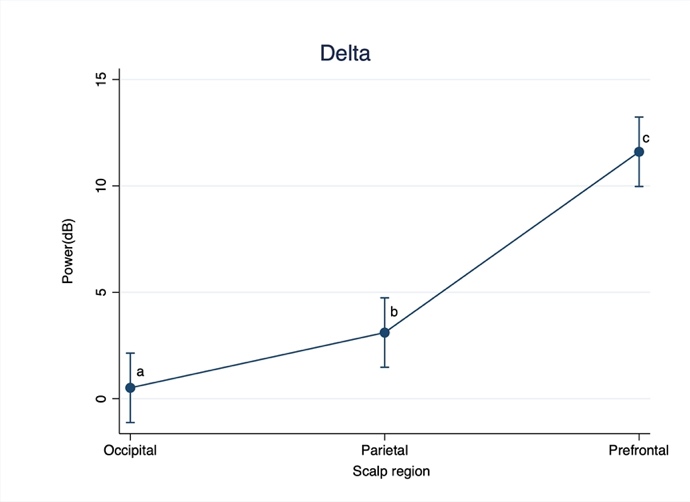**  1) Occipital vs. Parietal (D = 2.60 dB, p = 0.002)  2) Occipital vs. Prefrontal (D =11.09 dB, p < 0.001)  3) Parietal vs. Prefrontal (D =8.50 dB, p < 0.001) |
| --- | --- |
| **C**  **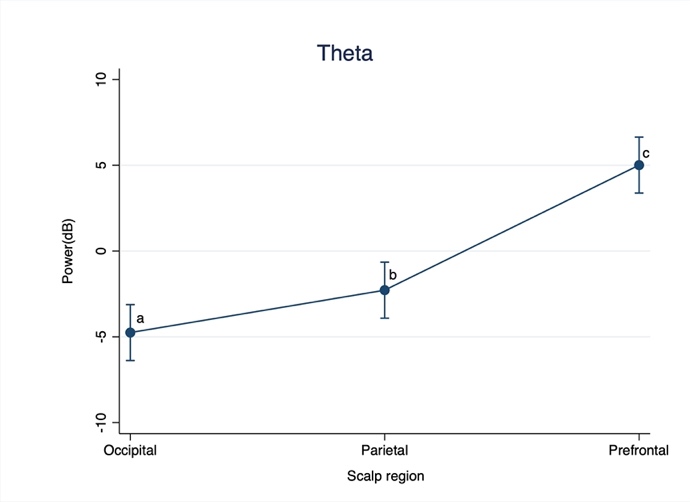**  1) Occipital vs. Parietal (D =2.47 dB, p = 0.003)  2) Occipital vs. Prefrontal (D =9.76 dB, p < 0.001)  3) Parietal vs. Prefrontal (D =7.29 dB, p < 0.001) | **D**  **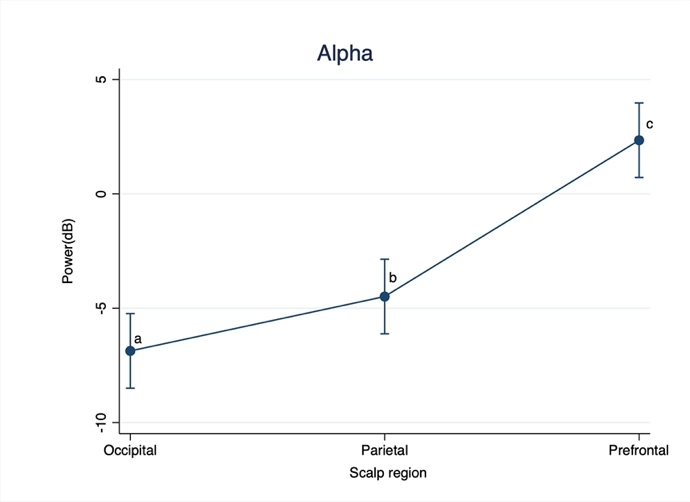**  1) Occipital vs. Parietal (D = 2.38 dB, p = 0.004)  2) Occipital vs. Prefrontal (D = 9.21 dB, p < 0.001)  3) Parietal vs. Prefrontal (D = 6.83 dB, p < 0.001) |
| **E**  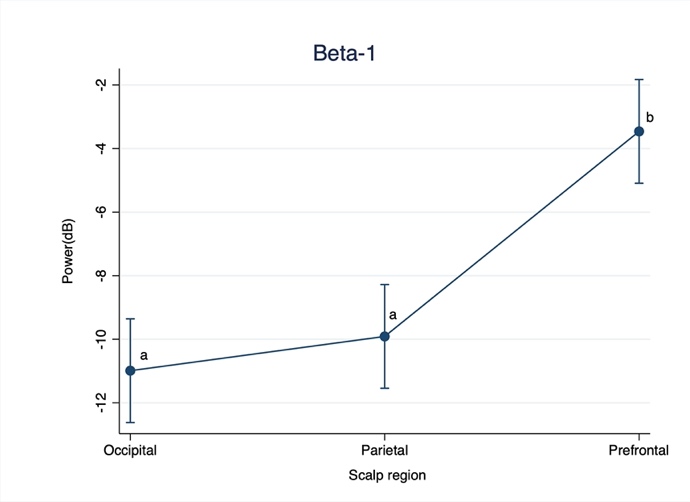  1) Occipital vs. Prefrontal (D =7.52 dB, p < 0.001)  2) Parietal vs. Prefrontal (D = 6.45 dB, p < 0.001) | 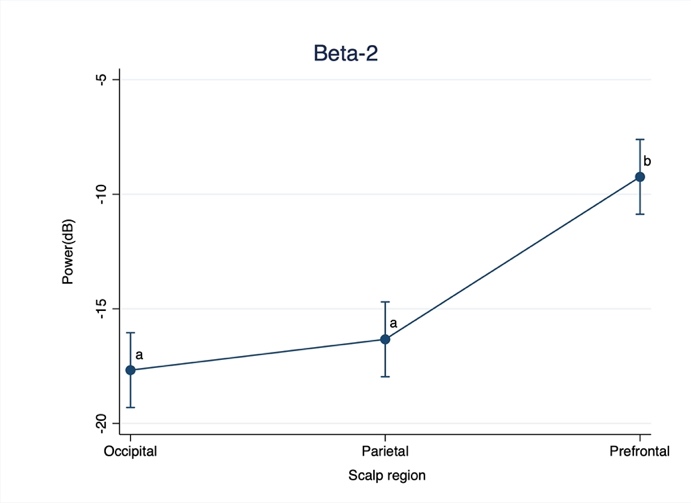**F**  1) Occipital vs. Prefrontal (D = 8.44 dB, p < 0.001)  2) Parietal vs. Prefrontal (D = 7.09 dB, p < 0.001) |
