# Supplementary material for: Spatio-temporal electroencephalographic power distribution in experimental pigs receiving propofol
Source: PLoS One. 2024 May 14;19(5):e0303146. doi: 10.1371/journal.pone.0303146 (PMC11093367; doi:10.1371/journal.pone.0303146)
Supplement: S2 Appendix — For statistical analysis, a mixed model was used with regions as fixed effects and pigs as random effects. Statistical significance (p < 0.01) is indicated with letters: Equal letters between two mean values indicate the absence of statistically significant difference. D = difference between EEG power values. (DOCX) [file pone.0303146.s002.docx]

| 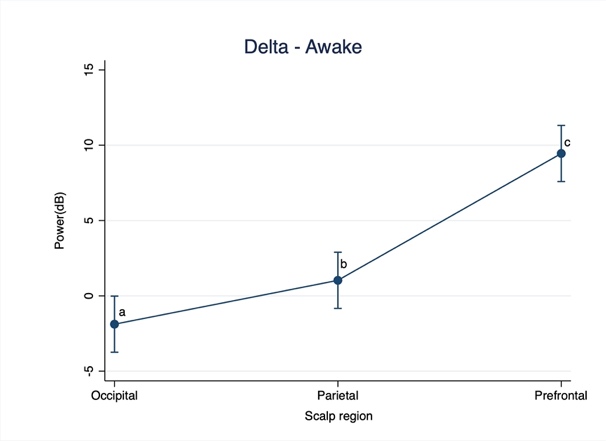 | Occipital vs. Parietal: D = 2.91, p = 0.005  Occipital vs. Prefrontal: D = 11.3, p < 0.001  Parietal vs. Prefrontal: D = 8.72, p < 0.0001 |
| --- | --- |
| 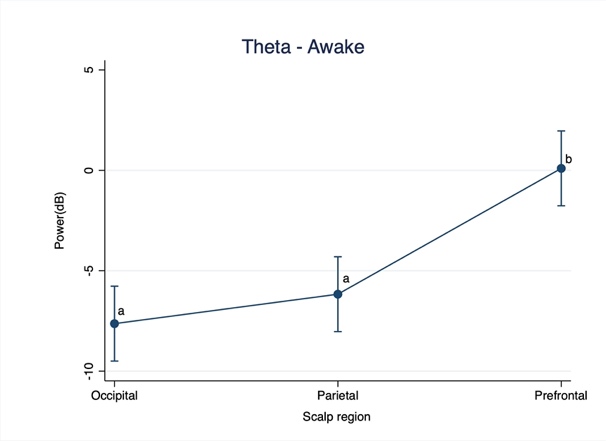 | Occipital vs. Prefrontal: D = 7.73, p < 0.001  Parietal vs. Prefrontal: D = 6.27, p < 0.0001 |
| 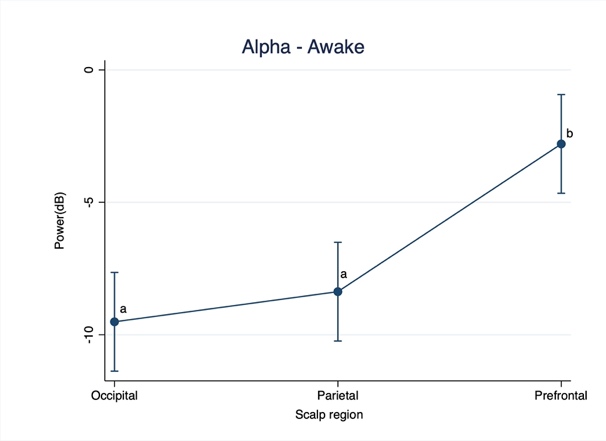 | Occipital vs. Prefrontal: D = 6.72, p < 0.001  Parietal vs. Prefrontal: D = 5.58, p < 0.0001 |
| 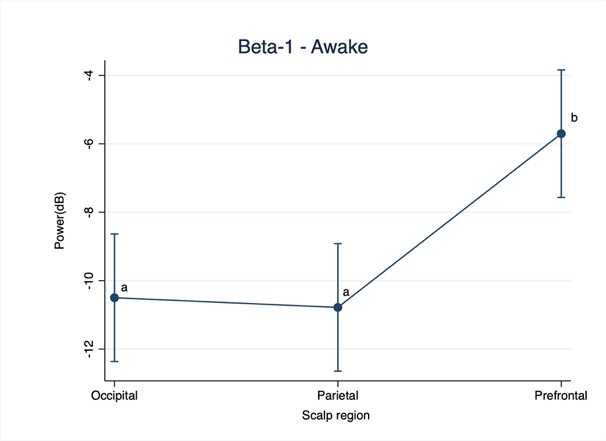 | Occipital vs. Prefrontal: D = 4.80, p < 0.001  Parietal vs. Prefrontal: D = 5.08, p < 0.0001 |
| 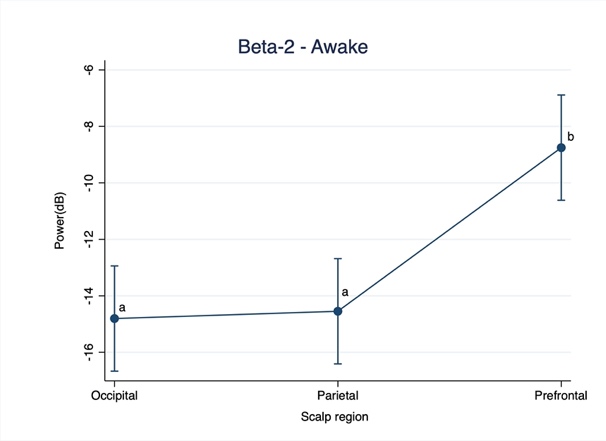 | Occipital vs. Prefrontal: D = 6.05, p < 0.001  Parietal vs. Prefrontal: D = 5.79, p < 0.0001 |

| 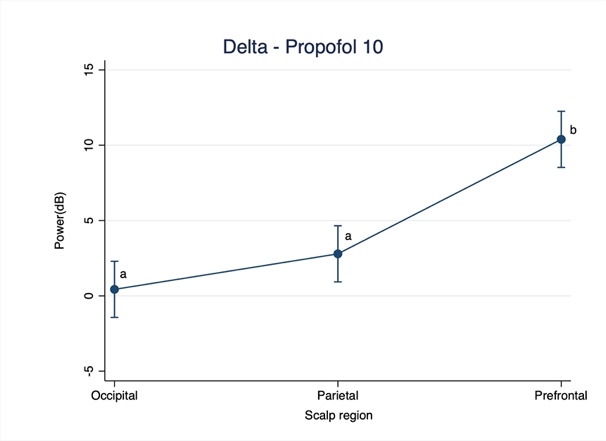 | Occipital vs. Prefrontal: D = 9.96, p < 0.001  Parietal vs. Prefrontal: D = 7.60, p < 0.0001 |
| --- | --- |
| 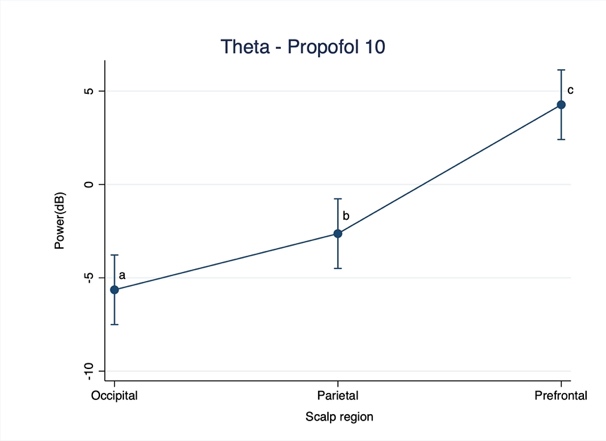 | Occipital vs. Parietal: D = 3.01, p = 0.004  Occipital vs. Prefrontal: D = 9.91, p < 0.001  Parietal vs. Prefrontal: D = 6.90, p < 0.0001 |
| 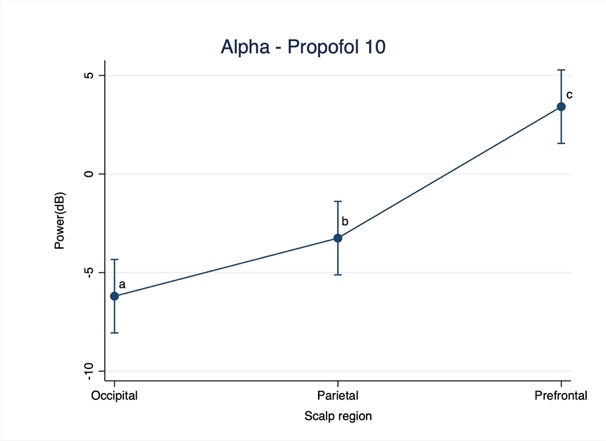 | Occipital vs. Parietal: D = 2.95, p = 0.004  Occipital vs. Prefrontal: D = 9.61, p < 0.001  Parietal vs. Prefrontal: D = 6.67, p < 0.0001 |
| 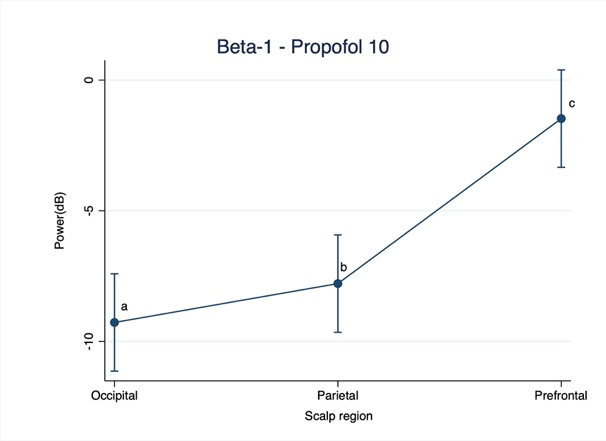 | Occipital vs. Prefrontal: D = 7.80, p < 0.001  Parietal vs. Prefrontal: D = 6.31, p < 0.0001 |
| 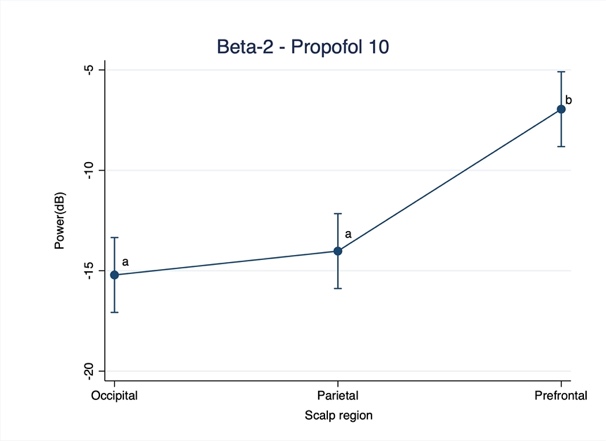 | Occipital vs. Prefrontal: D = 8.26, p < 0.001  Parietal vs. Prefrontal: D = 7.07, p < 0.0001 |

| 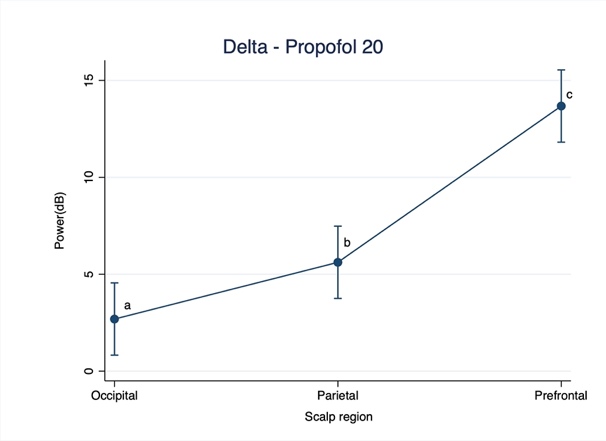 | Occipital vs. Parietal: D = 2.93, p = 0.005  Occipital vs. Prefrontal: D = 10.99, p < 0.001  Parietal vs. Prefrontal: D = 8.06, p < 0.0001 |
| --- | --- |
| 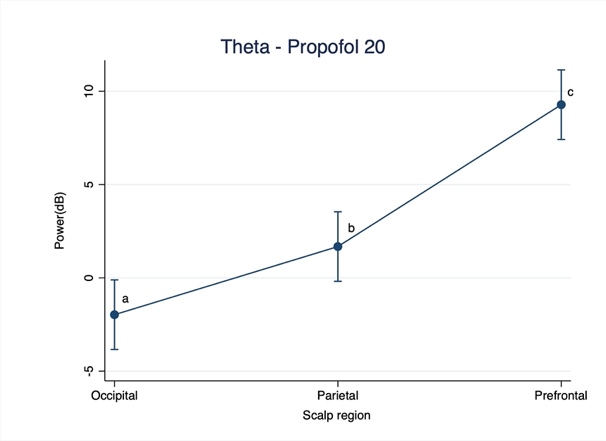 | Occipital vs. Parietal: D = 3.65, p < 0.001  Occipital vs. Prefrontal: D = 11.25, p < 0.001  Parietal vs. Prefrontal: D = 7.60, p < 0.0001 |
| 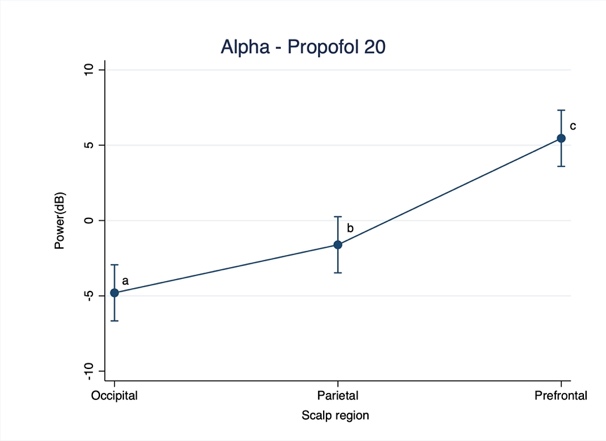 | Occipital vs. Parietal: D = 3.19, p = 0.002  Occipital vs. Prefrontal: D = 10.26, p < 0.001  Parietal vs. Prefrontal: D = 7.07, p < 0.0001 |
| 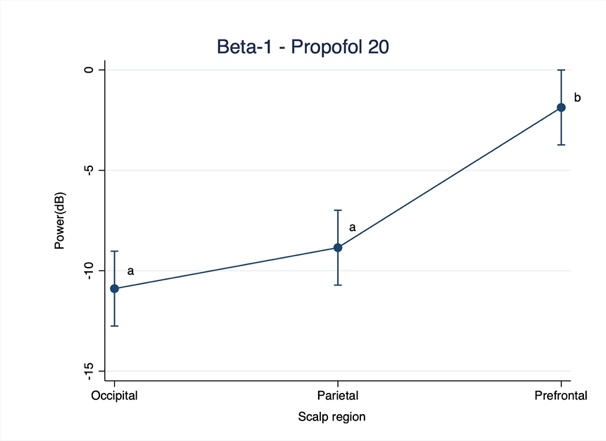 | Occipital vs. Prefrontal: D = 9.02, p < 0.001  Parietal vs. Prefrontal: D = 6.98, p < 0.0001 |
| 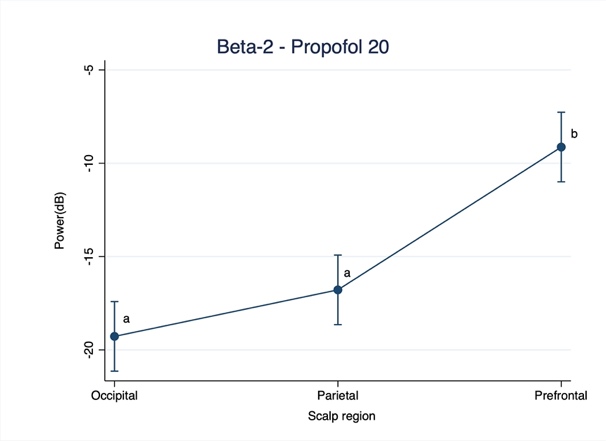 | Occipital vs. Prefrontal: D = 10.14, p < 0.001  Parietal vs. Prefrontal: D = 7.65, p < 0.0001 |

| 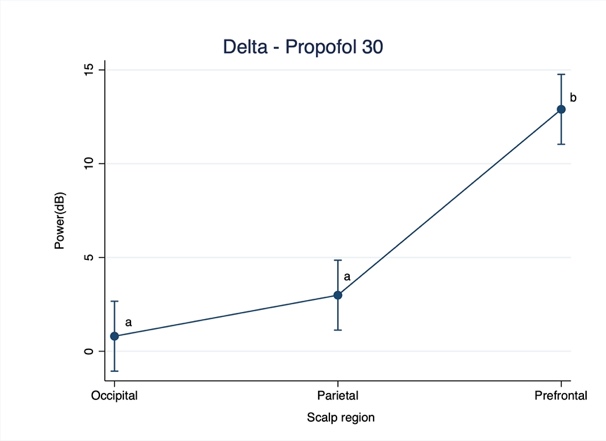 | Occipital vs. Prefrontal: D = 12.10, p < 0.001  Parietal vs. Prefrontal: D = 9.91, p < 0.0001 |
| --- | --- |
| 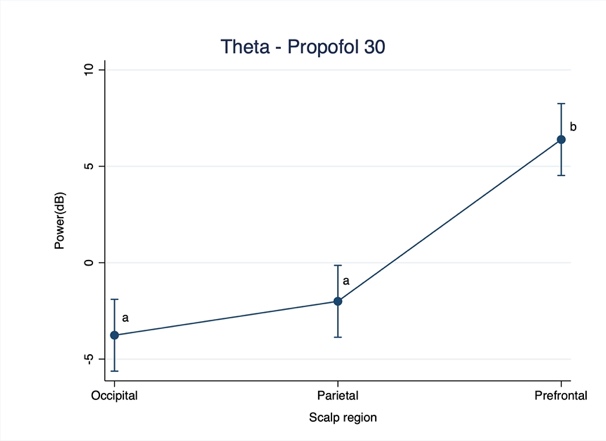 | Occipital vs. Prefrontal: D = 10.15, p < 0.001  Parietal vs. Prefrontal: D = 8.39, p < 0.0001 |
| 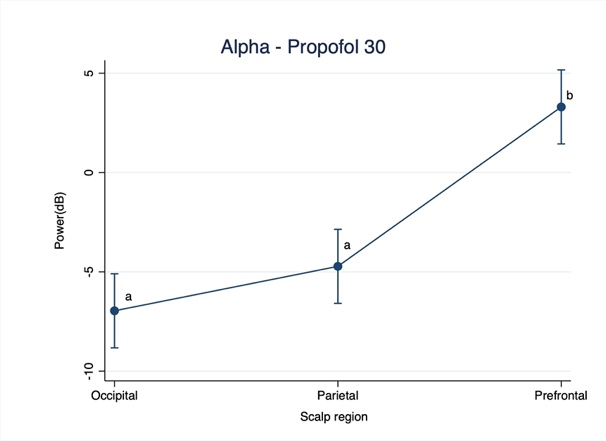 | Occipital vs. Prefrontal: D = 10.26, p < 0.001  Parietal vs. Prefrontal: D = 8.03, p < 0.0001 |
| 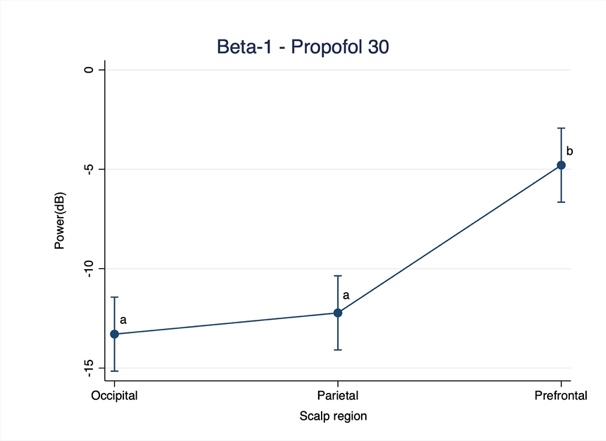 | Occipital vs. Prefrontal: D = 8.50, p < 0.001  Parietal vs. Prefrontal: D = 7.43, p < 0.0001 |
| 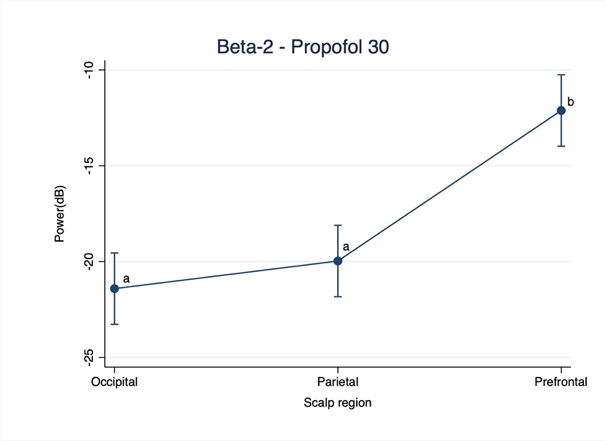 | Occipital vs. Prefrontal: D = 9.29, p < 0.001  Parietal vs. Prefrontal: D = 7.85, p < 0.0001 |
