# Supplementary figures and images for: Spatio-temporal electroencephalographic power distribution in experimental pigs receiving propofol
Source: PLoS One. 2024 May 14;19(5):e0303146. doi: 10.1371/journal.pone.0303146 (PMC11093367; doi:10.1371/journal.pone.0303146)

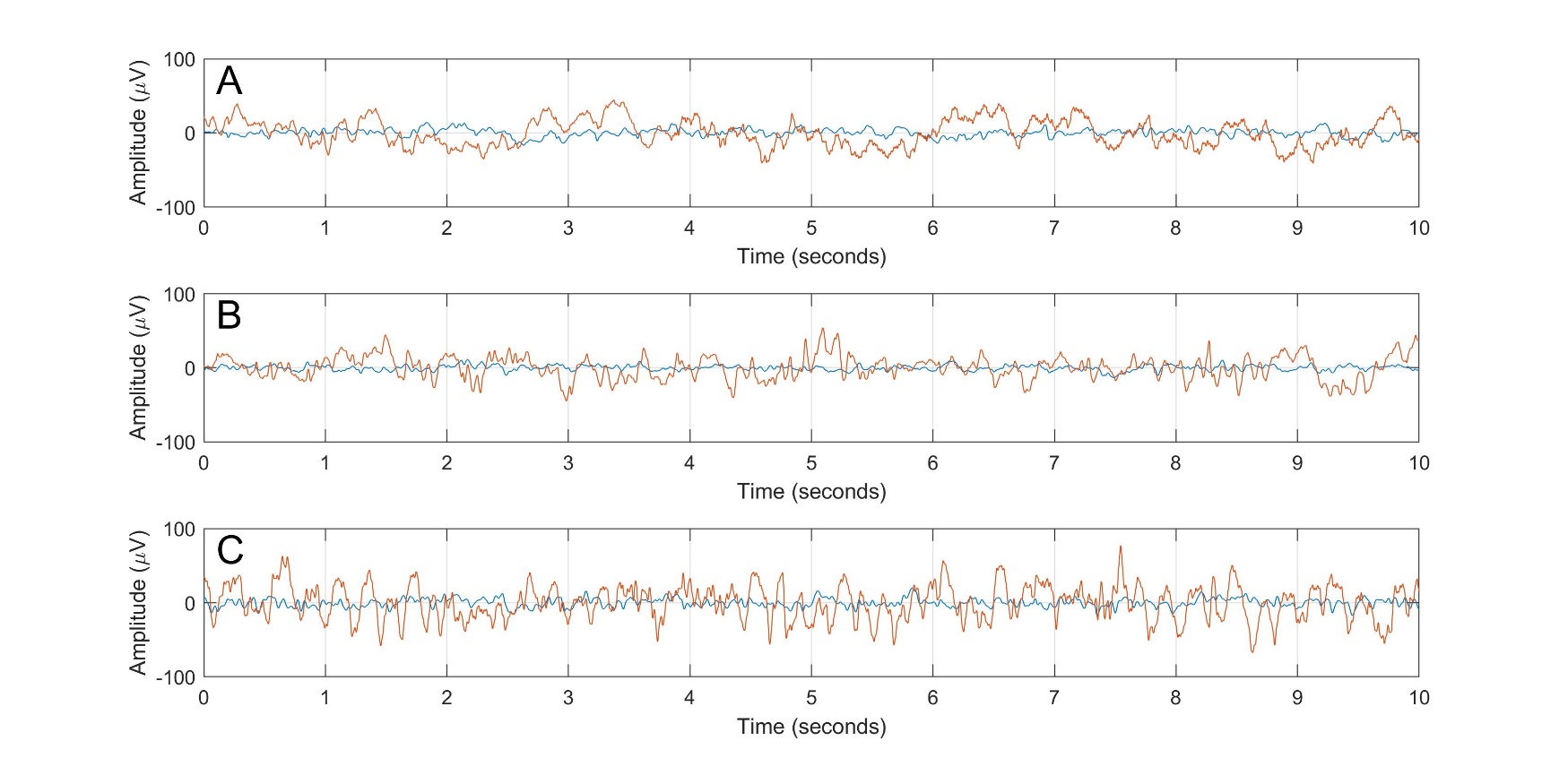

Supplement: S3 Appendix — (DOCX) [file pone.0303146.s003.docx]

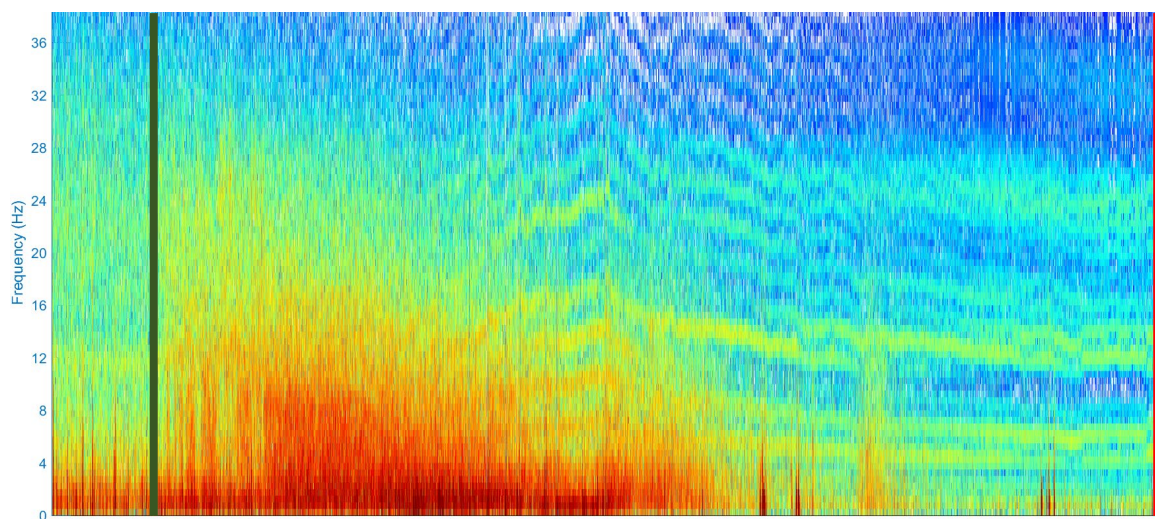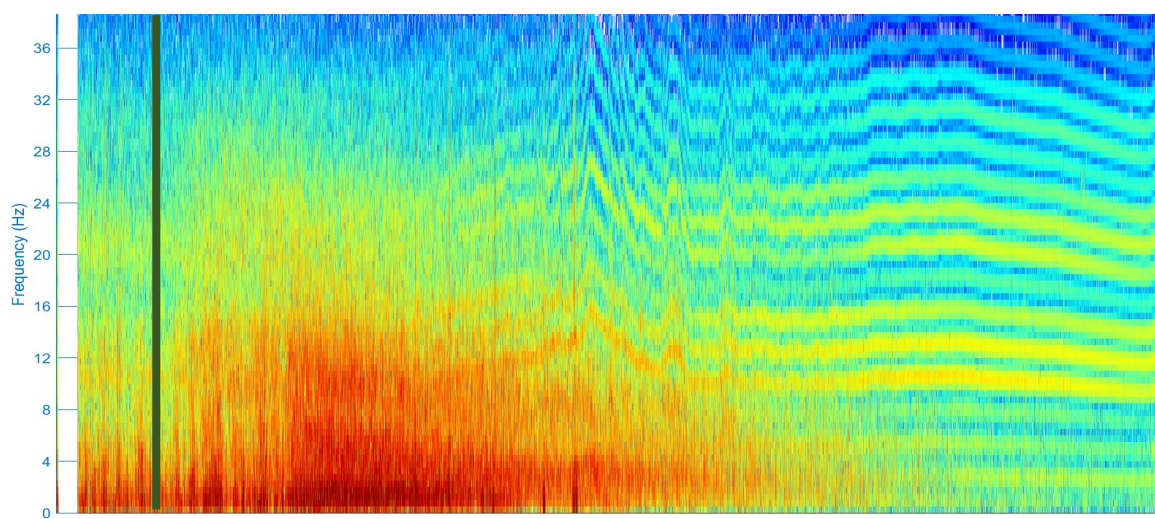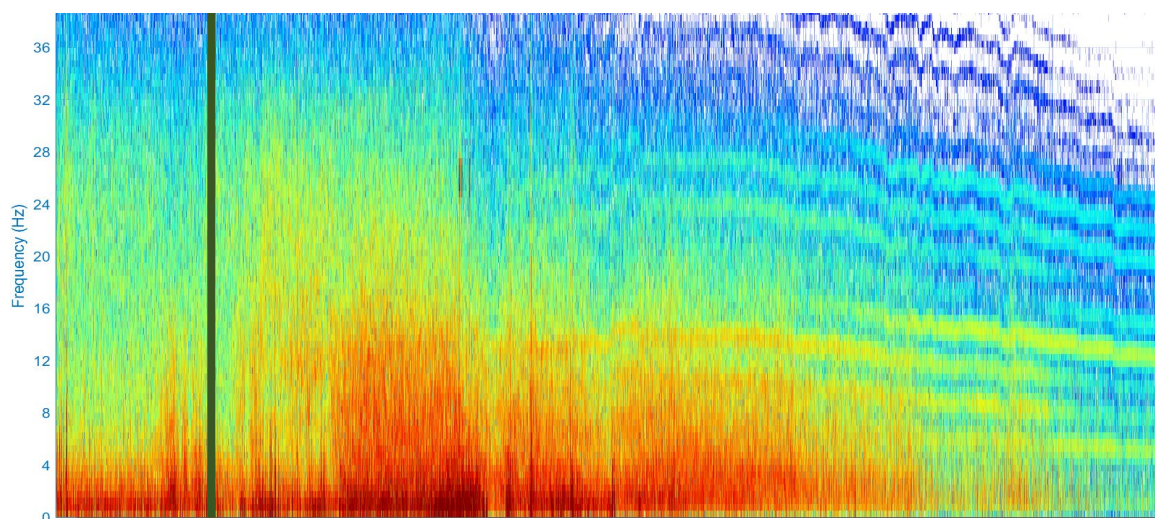

Time

Supplement: S4 Appendix — The signal has been recorded from the right prefrontal electrode. Green vertical line: Start of propofol infusion; red vertical line: End of propofol infusion. (PDF) [file pone.0303146.s004.pdf]
