# Supplementary material for: Spatio-temporal electroencephalographic power distribution in experimental pigs receiving propofol
Source: PLoS One. 2024 May 14;19(5):e0303146. doi: 10.1371/journal.pone.0303146 (PMC11093367; doi:10.1371/journal.pone.0303146)
Supplement: S5 Appendix — (DOCX) [file pone.0303146.s005.docx]

|  | M | N | O | P | Q | R |
| --- | --- | --- | --- | --- | --- | --- |
| **Awake** | 9.458415 | 13.32647 | 10.0721 | 9.986914 | 10.68918 | 10.05642 |
| **Propofol 10** | 9.148961 | 13.71218 | 11.493 | 8.664937 | 7.135191 | 9.024752 |
| **Propofol 20** | 9.682245 | 13.35437 | 13.04908 | 9.640607 | 11.76814 | 10.22603 |
| **Propofol 30** | 11.01028 | 13.46943 | 11.11223 | 11.37779 | 9.958368 | 13.75719 |

**Theta**

**Delta**

|  | M | N | O | P | Q | R |
| --- | --- | --- | --- | --- | --- | --- |
| **Awake** | 3.45986 | 11.27407 | 7.244772 | 9.24696 | 6.711209 | 9.5681 |
| **Propofol 10** | 8.102381 | 12.50149 | 11.4123 | 8.762604 | 7.720327 | 10.6202 |
| **Propofol 20** | 8.53342 | 12.65262 | 11.50698 | 13.3533 | 9.467357 | 12.92897 |
| **Propofol 30** | 7.233157 | 11.83461 | 9.008714 | 9.776597 | 10.14232 | 13.3303 |

**Alpha**

|  | M | N | O | P | Q | R |
| --- | --- | --- | --- | --- | --- | --- |
| **Awake** | 2.7880683 | 8.8022215 | 5.5487869 | 7.7717901 | 6.5418318 | 9.0075191 |
| **Propofol 10** | 7.8659314 | 12.341373 | 10.078072 | 8.0628147 | 7.2117084 | 11.968623 |
| **Propofol 20** | 6.5195258 | 11.704003 | 11.94475 | 8.5445806 | 8.7481367 | 13.897912 |
| **Propofol 30** | 7.3966233 | 12.364097 | 11.565291 | 10.18746 | 6.0151397 | 12.424636 |

**Beta**

|  | M | N | O | P | Q | R |
| --- | --- | --- | --- | --- | --- | --- |
| **Awake** | 2.883427 | 7.078709 | 4.301465 | 5.607547 | 5.767321 | 5.331938 |
| **Propofol 10** | 7.028865 | 11.94589 | 9.437398 | 3.891287 | 6.407562 | 11.44272 |
| **Propofol 20** | 7.044656 | 12.24866 | 9.34676 | 8.263541 | 6.96051 | 11.31502 |
| **Propofol 30** | 5.5365 | 12.35254 | 8.684942 | 9.222537 | 4.635149 | 12.69137 |

**Gamma**

|  | M | N | O | P | Q | R |
| --- | --- | --- | --- | --- | --- | --- |
| **Awake** | 5.253898 | 9.138222 | 6.972733 | 5.803298 | 6.330132 | 3.8868 |
| **Propofol 10** | 7.084428 | 11.79637 | 10.8427 | 3.01267 | 6.769072 | 11.50302 |
| **Propofol 20** | 8.582603 | 12.72725 | 9.493809 | 10.35761 | 7.800603 | 12.23085 |
| **Propofol 30** | 6.052905 | 13.05991 | 8.655321 | 9.551127 | 4.35024 | 14.01817 |
